# Quality evaluation of scar management information on TikTok: a cross-sectional study

**DOI:** 10.3389/fpubh.2026.1709429

**Published:** 2026-02-20

**Authors:** Ming Wang, Yi-Jing Jin, Feng Xu

**Affiliations:** 1 Department of Plastic and Reconstructive Surgery, Shanghai Ninth People's Hospital, Shanghai Jiao Tong University, Shanghai, China; 2 Department of Vascular Surgery, Shanghai Ninth People's Hospital, Shanghai Jiao Tong University, Shanghai, China; 3 Department of Cardiovascular Medicine, Hainan West Central Hospital, Hainan, China

**Keywords:** health information quality, media, scar management, social media, TikTok

## Abstract

**Background:**

Social media platforms like TikTok significantly influence health behaviors, yet the quality of scar management content remains under-evaluated. This study analyzes the quality, reliability, and actionability of scar management information on TikTok and examines the relationship between content quality and user engagement.

**Methods:**

A cross-sectional analysis of the 100 most-liked scar management videos was conducted. Two independent raters evaluated videos using mDISCERN, JAMA benchmark criteria, PEMAT-A/V, and the Global Quality Score (GQS). Creators were categorized as healthcare professionals (HCPs), content creators, or general users.

**Results:**

Healthcare professionals produced higher-quality content (GQS: 3.45 vs. 2.15 for creators; *p* < 0.001) with significantly better reliability and actionability. However, an “engagement paradox” was observed: lower-quality videos from non-professionals garnered significantly higher engagement (likes, shares) than evidence-based professional content. Misinformation was prevalent in 46.2% of content creator videos.

**Conclusion:**

A structural disconnect exists on TikTok where accurate medical advice is overshadowed by algorithmically favored, visually stimulating, but often misleading content. Addressing this public health risk requires platform-level algorithmic adjustments and enhanced digital strategies from medical professionals to compete in the attention economy.

## Introduction

Scars, the inevitable outcome of wound healing following surgery, trauma, or burns, represent one of the most common reasons for patient consultation in plastic and reconstructive surgery. The burden of scarring extends far beyond cosmetic concerns, often imposing significant physical and psychosocial consequences. Pathological scars, such as hypertrophic and keloid scars, can cause pain, pruritus, and functional limitation due to contractures, while aesthetically displeasing scars can lead to anxiety, depression, and a diminished quality of life (1). Effective, evidence-based scar management—encompassing a range of interventions from topical treatments and pressure therapy to laser procedures and surgical revision—is therefore crucial for optimizing both functional and psychological patient outcomes (2).

In the digital age, social media platforms have become increasingly important sources of health information, particularly among younger demographics. TikTok, a short-form video platform with over one billion active users globally, has emerged as a significant channel for health information dissemination (3). The platform's algorithm-driven content delivery and highly engaging, visual format make it particularly influential in shaping health-related perceptions and behaviors, especially in visually-driven fields like dermatology and plastic surgery. Research examining health information on social media has revealed significant variability in content quality and accuracy (4). While some healthcare professionals use these platforms to disseminate evidence-based information, concerns remain about the potential spread of misinformation and its impact on public health, particularly from creators without medical training who can reach vast audiences (5).

Scar-specific information on social media presents unique challenges due to the heavy commercialization of the scar treatment market and the complexity of matching treatments to specific scar types. Misinformation can have profound consequences: promoting ineffective or unproven “miracle” products can lead to financial loss and delay access to effective medical care, while improper advice could potentially worsen scarring or cause adverse skin reactions. The emotionally charged nature of dealing with scars also makes individuals vulnerable to compelling personal testimonials and persuasive marketing over objective medical facts. Despite these concerns, the specific landscape of scar management on TikTok remains underexplored. Previous studies have examined dermatological content generally, but few have analyzed the specific disconnect between information quality and user engagement in the context of scar treatment—a field heavily saturated with commercial “miracle” products.

To understand this dynamic, this study draws on the Elaboration Likelihood Model (ELM). ELM suggests that in media-rich environments, users often process information via “peripheral cues” (e.g., before-and-after visuals, creator charisma) rather than “central processing” of factual accuracy. This framework helps explain why users may engage more with visually compelling but medically inaccurate content. Furthermore, the Extended Parallel Process Model (EPPM) offers insight into how users manage the fear of permanent scarring, potentially driving them toward “miracle cures” that promise immediate relief.

Therefore, this study aims to: (1) systematically evaluate the quality of scar management content on TikTok using validated instruments; and (2) apply the ELM framework to analyze the paradox between content quality and user engagement.

## Methods

### Study design and data collection

This investigation was structured as a cross-sectional content analysis of scar management videos on the TikTok platform. To establish a standardized, algorithm-neutral baseline for content retrieval, we utilized a newly created TikTok account with its profile set to a 25-year-old user in the United States. This account had no associated viewing or interaction history. The entire data gathering process was executed within a specific timeframe, from March 1st to March 7th, 2025, following a pre-defined and consistent protocol.

### Video selection criteria

A purposive sampling strategy was employed to identify the most influential content within our area of focus. Content was identified through a systematic query of five prominent English-language hashtags: scartreatment, scarremoval, keloid, hypertrophicscar, and scartherapy. To focus on content with the highest public visibility and potential for impact, the search results from each query were sorted by the platform's “most-liked” metric. From these results, the top 100 unique videos that conformed to our eligibility requirements were compiled to form the study's final sample.

Eligibility was determined by a strict set of criteria. Videos were required to be in English, have a duration between 15 s and 10 min, and have a primary theme centered on scar management health information. Content was disqualified if it was a duplicate, irrelevant to cutaneous scars, primarily a personal story devoid of educational advice, or not in English. Following selection, a standardized coding sheet was used to capture video attributes, including duration, and user engagement data (likes, comments, shares), as well as creator details such as username, follower count, and professional credentials listed in their profile. This information was crucial for the subsequent classification of information sources.

### Video analysis and coding

Recognizing that information quality is a multifaceted construct, our evaluation protocol integrated four validated instruments to provide a robust and holistic assessment. We employed a multi-dimensional framework to assess video quality. Reliability and clarity were evaluated via the modified DISCERN (mDISCERN) tool, while the JAMA benchmark criteria served to gauge source transparency. Additionally, to measure how easily users could understand and act upon the information, we applied the Patient Education Materials Assessment Tool (PEMAT-A/V). Finally, the Global Quality Score (GQS) provided a holistic rating of overall educational utility.

The entire coding process was conducted by two trained researchers who independently evaluated all 100 videos. To ensure consistency, a consensus meeting was held to resolve any scoring discrepancies, with a third senior researcher arbitrating if needed. The inter-rater reliability was strong, with a calculated Cohen's kappa (κ) of 0.85 for the GQS scores, indicating high agreement between the two independent raters.

### Statistical analysis

All quantitative data were analyzed using SPSS Statistics (Version 28.0). Initial data exploration was conducted using descriptive statistics to outline the general characteristics of the video sample. Given the skewed nature of the data, particularly the engagement metrics and quality scores, we exclusively used non-parametric statistical methods for hypothesis testing. Differences in median scores across the defined creator categories were assessed using the Kruskal-Wallis *H* test, with appropriate *post-hoc* analyses conducted when significant variations were detected. The association btween content quality scores and user engagement levels was quantified using Spearman's rank correlation coefficient. A threshold for statistical significance was established at *p* < 0.05 for all analyses.

## Results

**Figure 1:**
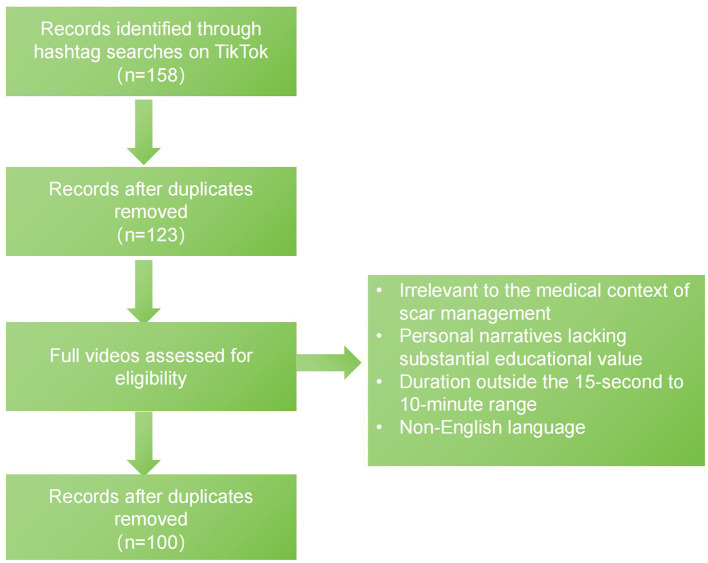
Flowchart of the video selection process.

A total of 100 unique TikTok videos met the inclusion criteria and were included in the final analysis. The detailed video selection process is illustrated in Figure 1. The sample exhibited wide variability in video duration, creator characteristics, user engagement metrics, and overall content quality. Table 1 provides a comprehensive summary of the characteristics of the analyzed videos.

**Table 1:** Characteristics of TikTok videos related to scar management (*n* = 100).

**Characteristic**	**Healthcare professionals (*n* = 31)**	**Content creators (*n* = 52)**	**General users (*n* = 17)**	**Overall (*n* = 100)**	***p*-value**
**Video Duration (seconds), median [IQR]**	68 [45–90]	55 [35–75]	48 [30–62]	58 [38–81]	0.08
**User engagement metrics, median [IQR]**					
Likes	15,100 [8,500–28,000]	65,800 [30,100–150,500]	22,400 [11,000–41,000]	38,500 [14,000–98,000]	<0.001
Comments	250 [110–450]	980 [450–2,100]	310 [150–550]	550 [200–1,200]	<0.001
Shares	890 [400–1,500]	4,200 [1,800–9,500]	1,100 [550–2,000]	2,150 [800–6,500]	<0.001
**Creator characteristics**					
Follower count, median [IQR]	85k [25k−250k]	550k [150k−1.5M]	15k [5k−50k]	180k [30k−800k]	<0.001
Verified profile, *n* (%)	18 (58.1%)	24 (46.2%)	1 (5.9%)	43 (43.0%)	<0.001
Disclosed credentials, *n* (%)	30 (96.8%)	3 (5.8%)	0 (0%)	33 (33.0%)	<0.001

IQR, interquartile range.

*p*-values calculated using Kruskal-Wallis H test for continuous variables and Chi-squared test for categorical variables.

Video creators were categorized into three groups: healthcare professionals (31%), content creators (e.g., beauty influencers) (52%), and general users (17%). While healthcare professionals often had fewer followers than top-tier content creators, they had significantly higher rates of profile verification and explicit disclosure of their medical credentials. Content creators garnered the highest median engagement metrics, receiving significantly more likes, comments, and shares than both healthcare professionals and general users (*p* < 0.001). This engagement advantage for content creators remained even after accounting for follower count, suggesting a more effective use of platform-specific engagement strategies.

The assessment of content quality revealed profound and statistically significant disparities across the three creator types, with videos produced by healthcare professionals consistently achieving higher scores on all validated metrics.

Regarding the Global Quality Score (GQS), videos produced by medical experts averaged 3.45 ± 0.72, with 83.9% meeting the quality threshold of three. This superiority was even more pronounced in reliability assessments (mDISCERN), where professionals consistently provided balanced information (mean 3.51 ± 0.65). In terms of actionability (PEMAT-A/V), a significant gap emerged: while professionals scored 81.5%, content creators and general users lagged behind at 55.4% and 41.2%, respectively (*p* < 0.001). Source transparency analysis similarly favored professionals, who nearly universally identified authorship (100%), standing in sharp contrast to the minimal disclosure rates among general users.

The JAMA benchmark criteria, which measure source transparency, revealed the most striking differences. Healthcare professionals nearly universally identified authorship (100.0%) and provided attribution for sources in 61.3% of videos. This sharply contrasted with content creators (authorship 19.2%, attribution 5.8%) and general users (authorship 5.9%, attribution 0%) (*p* < 0.001) (Table 2).

**Table 2:** Quality assessment scores across creator types.

**Quality assessment metric**	**Healthcare professionals (*n* = 31)**	**Content creators (*n* = 52)**	**General users (*n* = 17)**	***p*-value**
**Global quality score (GQS), mean** **±SD (1–5 scale)**	3.45 ± 0.72	2.15 ± 0.88	1.76 ± 0.68	<0.001
**Modified DISCERN (mDISCERN), mean** **±SD (0–5 scale)**	3.51 ± 0.65	1.98 ± 0.91	1.65 ± 0.79	<0.001
**PEMAT-A/V Understandability score, % mean**	88.20%	81.40%	79.50%	0.03
**PEMAT-A/V Actionability score, % mean**	81.50%	55.40%	41.20%	<0.001
**JAMA benchmark criteria score, mean** **±SD (0–4 scale)**	3.23 ± 0.81	0.44 ± 0.65	0.12 ± 0.33	<0.001
**Met authorship criterion**, ***n*** **(%)**	31 (100.0%)	10 (19.2%)	1 (5.9%)	<0.001
**Met attribution criterion**, ***n*** **(%)**	19 (61.3%)	3 (5.8%)	0 (0%)	<0.001

SD, standard deviation; GQS, Global Quality Score; PEMAT-A/V, Patient Education Materials Assessment Tool for Audiovisual content; JAMA, Journal of the American Medical Association.

*p*-values calculated using Kruskal-Wallis *H* test or Chi-squared test.

Content analysis identified a strong thematic focus on specific products and cosmetic procedures (72% of all videos) over foundational education on scar biology and prevention. Only 17% of videos explained concepts like the scar maturation process or the importance of proper wound care to minimize scarring. Healthcare professionals were more likely to discuss evidence-based foundational treatments like silicone sheeting and pressure therapy (58.1% of their videos) compared to content creators (15.4%) and general users (5.9%). In contrast, content creators and general users disproportionately focused on “miracle” creams and unproven DIY home remedies (Table 3).

**Table 3:** Comprehensive content analysis of scar management-related information.

**Video content theme**	**Healthcare professionals (*n* = 31)**	**Content creators (*n* = 52)**	**General users (*n* = 17)**	**Overall (*n* = 100)**
**Primary focus of video**				
Specific product/cosmetic procedure	15 (48.4%)	42 (80.8%)	15 (88.2%)	72 (72.0%)
Foundational scar education	12 (38.7%)	4 (7.7%)	1 (5.9%)	17 (17.0%)
Prevention/wound care	9 (29.0%)	3 (5.8%)	0 (0%)	12 (12.0%)
**Specific treatments mentioned**				
Evidence-Based (e.g., Silicone, Pressure)	18 (58.1%)	8 (15.4%)	1 (5.9%)	27 (27.0%)
Unproven “Miracle” creams/serums	7 (22.6%)	35 (67.3%)	12 (70.6%)	54 (54.0%)
DIY home remedies	2 (6.5%)	22 (42.3%)	9 (52.9%)	33 (33.0%)

Data presented as *n* (%).

Categories are not mutually exclusive; a single video could contain multiple themes.

Alarmingly, misinformation permeated over one-third (36%) of the entire sample (Table 4). While healthcare professionals largely maintained accuracy (9.7% misinformation rate), non-professional creators were significant sources of error, with nearly half of their content containing medical inaccuracies (46.2% for creators and 52.9% for general users). Qualitative analysis revealed that these inaccuracies primarily manifested as the promotion of scientifically baseless products, potentially harmful DIY regimens (e.g., abrasive scrubs), and exaggerated treatment outcomes. Qualitative analysis of the high-engagement/low-quality videos revealed a heavy reliance on visual shock value. For example, one video (2.1 million likes) by a general user recommended scrubbing fresh hypertrophic scars with undiluted lemon juice—a practice that can cause severe irritation—accompanied by highly filtered “after” photos claiming total scar removal in three days. Such content utilizes the “peripheral cues” described in the ELM to bypass critical evaluation.

**Table 4:** Misinformation analysis and educational value assessment.

**Assessment category**	**Healthcare professionals (*n* = 31)**	**Content creators (*n* = 52)**	**General users (*n* = 17)**	**Overall (*n* = 100)**
**Presence of misinformation**, ***n*** **(%)**	3 (9.7%)	24 (46.2%)	9 (52.9%)	36 (36.0%)
**Types of misinformation**, ***n*** **(%)**				
Promotion of unproven products	2 (6.5%)	21 (40.4%)	8 (47.1%)	31 (31.0%)
Recommendation of harmful DIY treatment	0 (0%)	11 (21.2%)	6 (35.3%)	17 (17.0%)
Misrepresentation of outcomes	1 (3.2%)	15 (28.8%)	5 (29.4%)	21 (21.0%)
**Primary video format**, ***n*** **(%)**				
Educational/didactic	25 (80.6%)	12 (23.1%)	2 (11.8%)	39 (39.0%)
Personal testimonial/before-after	6 (19.4%)	40 (76.9%)	15 (88.2%)	61 (61.0%)

Data presented as *n* (%).

Categories are not mutually exclusive.

Crucially, correlation analysis revealed no significant association between any of the content quality scores and user engagement metrics (GQS vs. likes: *r* = −0.05, *p* = 0.62; mDISCERN vs. comments: *r* = 0.09, *p* = 0.37). This indicates that higher-quality, more reliable medical information did not translate to greater user interaction. In fact, videos featuring dramatic “before and after” visuals and personal testimonials achieved significantly higher engagement (median likes: 45,200) than didactic, evidence-based educational videos (median likes: 15,100, *p* < 0.01), regardless of the accuracy of the information presented.

## Discussion

This study represents the first comprehensive evaluation of scar management information on TikTok, revealing a stark disconnect between content quality and user engagement. Our findings demonstrate that while healthcare professionals consistently produce higher-quality, more reliable, and actionable information, their content is significantly less visible and engaging than the often-misleading videos produced by non-professional content creators. This “engagement paradox,” coupled with a high prevalence of misinformation, highlights a critical public health challenge in the digital dissemination of dermatological and surgical aftercare advice.

The superior performance of healthcare professionals across all quality metrics—including GQS, mDISCERN, PEMAT-A/V, and JAMA criteria—is consistent with a growing body of literature evaluating health information on social media platforms like YouTube, Instagram, and TikTok itself (6–8). Healthcare professionals demonstrated a commitment to evidence-based medicine, transparency through source attribution, and responsible communication by providing actionable, safe advice. The deficits observed among content creators and general users—primarily a lack of source citation, promotion of unproven products, and oversimplified or inaccurate medical explanations—underscore the risks of a democratized information ecosystem where expertise is not a prerequisite for influence (9).

A particularly troubling finding is the complete absence of a positive correlation between content quality and user engagement. This “engagement paradox” aligns with the Elaboration Likelihood Model. On TikTok, the short-form, fast-paced nature of consumption favors the “peripheral route” of persuasion, where users prioritize superficial cues—such as dramatic music, emotional testimonials, and visual transformations—over the “central route” of cognitive scrutiny required for evidence-based medical advice (8, 10). Consequently, factual but dry medical content fails to trigger the algorithms that reward high-retention “entertainment” value. This phenomenon contributes to what the World Health Organization describes as an “infodemic”—an overabundance of information that makes it hard for people to find trustworthy sources (11). In the context of scar management, this infodemic is driven by the platform's algorithmic architecture, which amplifies engagement regardless of veracity, prioritizing sensationalism over scientific accuracy.

The thematic analysis revealed a significant content gap, with an overwhelming focus on commercial products and cosmetic procedures rather than foundational education on scar biology and prevention. This reflects the highly commercialized nature of the scar treatment market, where direct-to-consumer advertising and influencer marketing are rampant (12). This product-centric focus represents a missed opportunity for population-level health education, as proper initial wound care and an understanding of the scar maturation process are fundamental to achieving optimal outcomes (4).

The presence of misinformation in over one-third of all analyzed videos—and in nearly half of the videos from non-professionals—poses a direct risk to public health. The promotion of unproven “miracle” products can lead to significant financial waste and, more importantly, delay the use of effective, evidence-based treatments (13). Furthermore, the recommendation of potentially harmful DIY remedies could cause contact dermatitis, infection, or even worsen scarring (14). This finding highlights an urgent need for enhanced digital health literacy among consumers, equipping them with the skills to critically evaluate the credibility of online health sources (11).

This study has several limitations. Its cross-sectional design captures only a single point in time and cannot account for evolving content trends. The analysis was restricted to English-language videos, which limits the generalizability of our findings to TikTok's global user base. Our sampling strategy, which focused on the “most-liked” videos, is a limitation. While this approach captures the content with the highest public visibility and potential impact, it likely introduces a survivorship bias, excluding high-quality educational videos that failed to achieve algorithmic virality. Finally, our correlational analysis cannot establish causality between content features and engagement, as it does not control for confounders like creator follower count or the video's age.

Despite these limitations, our findings have clear implications for multiple stakeholders. Healthcare professionals and their organizations must move beyond passively creating accurate content and actively learn the language of the platform—using engaging visuals, storytelling, and clear calls-to-action—to compete in the digital attention economy. Platform developers have a responsibility to refine their algorithms to better distinguish and prioritize content from verified health experts, potentially through quality labels or badges, and to more aggressively de-platform harmful misinformation. Future research should explore the real-world impact of this content on patient behavior and clinical outcomes, conduct cross-platform comparisons, and design and test interventions aimed at improving the creation and visibility of high-quality health information on short-form video platforms.

## Conclusion

The landscape of scar management information on TikTok is characterized by a “quality-visibility inversion,” where inaccurate content thrives due to algorithmic prioritization of engagement over accuracy. This study confirms that while healthcare professionals provide the safest advice, they are losing the battle for attention. Addressing this is not merely a task for individual creators but a structural imperative for platform governance. Solutions must move beyond content creation to include algorithmic down-ranking of health misinformation and the implementation of verified “Health Expert” badges. Only through such systemic changes can social media transition from a source of medical noise to a reliable tool for patient empowerment.

## Data Availability

The original contributions presented in the study are included in the article/supplementary material, further inquiries can be directed to the corresponding author.
